# Adaptive optics ophthalmoscopy in retinitis pigmentosa (RP): Typical patterns

**DOI:** 10.1111/aos.15183

**Published:** 2022-05-25

**Authors:** Friederike C. Kortuem, Melanie Kempf, Laura Kuehlewein, Fadi Nasser, Constanze Kortuem, Michel Paques, Susanne Kohl, Marius Ueffing, Bernd Wissinger, Eberhart Zrenner, Katarina Stingl

**Affiliations:** ^1^ Center for Ophthalmology University Eye Hospital, University of Tuebingen Tuebingen Germany; ^2^ Center for Rare Eye Diseases University of Tuebingen Tuebingen Germany; ^3^ Institute for Ophthalmic Research, Center for Ophthalmology University of Tuebingen Tuebingen Germany; ^4^ Werner Reichardt Centre for Integrative Neuroscience (CIN) University of Tuebingen Tuebingen Germany; ^5^ Department of Ophthalmology Quinze‐Vingts Hospital, INSERM‐DHOS CIC Paris France

Editor,

We like to report on typical morphological findings in Retinitis pigmentosa (RP), based on 3 years clinical experience with the adaptive optics (AO) in the Clinics for Hereditary Retinal Degenerations at the Center for Ophthalmology, Tuebingen. We could establish five morphological patterns of AO that were repetitively observed in patients with RP using multimodal imaging on 174 patients with syndromic or non‐syndromic RP (in most of the cases genetically confirmed). These five patters are described as:

## Unspecific Atrophy

**Fig. 1:**
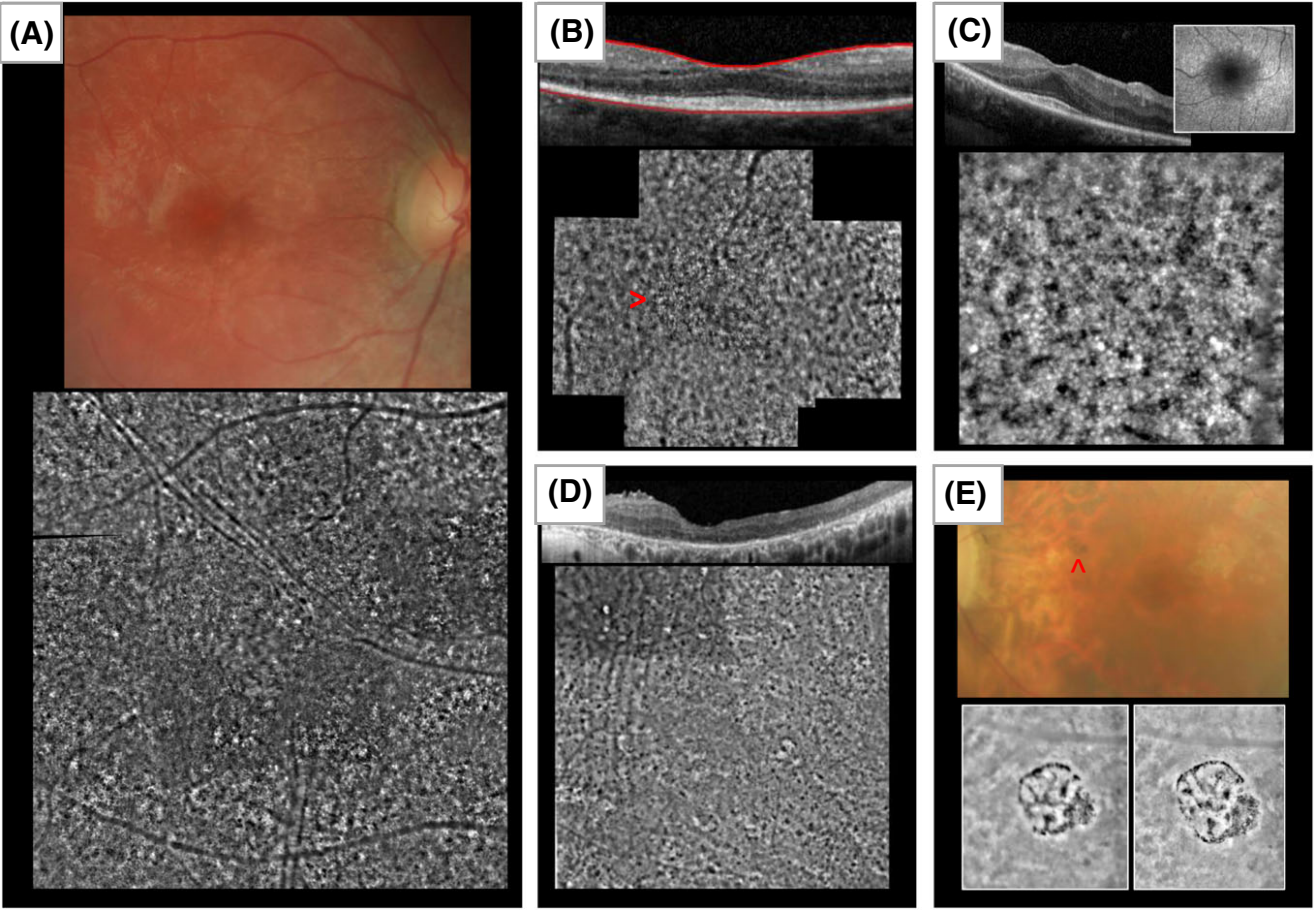
(A) Unspecific Atrophy: Fundus photograph, central montage of AO. The atrophy in the photograph appears less pronounced than in the AO image. (B) Central Visibility of Cones: OCT scan through foveal depression, central montage of AO images. Demarcation of the central photoreceptor area is visible, indicated with an arrow. (C) Puffy cones: OCT scan, magnified AO image. Left upper corner next to OCT scan: fundus autofluorescence image central without AO, there is a hyperfluorescent ring visible, corresponding to the area of ‘puffy’ cones. (D) Cheetah Pattern: OCT scan through foveal depression, central montage of AO image. The cheetah pattern is found throughout the macular area. (E) Regional atrophic pigment clumping: Fundus photograph, AO images of lesion indicated in the photo with red arrow – baseline examination and follow‐up after 9 months. Clumping has increased over time. [Colour figure can be viewed at wileyonlinelibrary.com]

In a large number of patients with RP, an unspecific atrophy (Fig. 1A) can be observed representing a late stage of photoreceptor degeneration. Unspecific atrophy pattern is not only characteristic for RP, but can be found in any photoreceptor degeneration. The unspecific atrophy on AO is characterized by dark patchy atrophic zones without clearly distinguishable cone mosaic and is often accompanied by epiretinal membranes, visible as folds on the epiretinal side, a frequent finding in RP patients.

## Central Visibility of Cones

Normally, due to an insufficient resolution the central cones of the fovea are not visible (Putnam, Hammer, Zhang, Merino, & Roorda, 2010). However, for RP the concentration of cone density is decreased even before changes in OCT become visible (Sun *et al*., 2016). We found that for some RP patients a cone mosaic pattern in the very centre of the macula could be observed on AO. The visible rather homogenous cone mosaic corresponds to the residual photoreceptors on OCT imaging and a good visual acuity. In our patients, the mosaic was comparable with parafoveal RP cone mosaics partly with dark patchy areas.

## Puffy Cones

This morphologically characteristic stage of photoreceptor degeneration can be found predominantly in rather younger patients with RP. The morphological change in the cone appearance can be described as ‘puffy’: They are larger in size. The borders of the cells do not appear clearly defined as in a normal retina, but swollen, whereas the reflectance is still given (Fig. 1C). Multimodal imaging shows that puffy cones are frequently found in areas of or close to the hyperfluorescent ring on fundus autofluorescence imaging, corresponding to the transition zone of healthy‐degenerated retina. OCT indicates that the sections of puffy cones are the areas of outer segment loss and/or ellipsoid zone loss with still preserved cell bodies. We hypothesize that puffy cones may represent an early stage of photoreceptor degeneration localized often at the borderline of degenerated and preserved retina. However, the cause of this abnormal reflectivity remains unknown.

## Cheetah Pattern

These broad relatively homogeneous areas of dark dot‐shaped atrophy mosaic (Fig. 1D) are typically observed in the *RPGR* phenotype, however, can be found also in other genotypes. OCT shows that the outer retina and the outer nuclear layer are barely visible corresponding to a complete loss of photoreceptors. Comparing the OCT with AO a diffuse ‘cheetah skin’ pattern might be a sign of homogeneous profound outer retinal atrophy and RPE loss. This correlates with the visibility of choroid vessels on fundus imaging. We found also that AO images might sometimes display the ‘cheetah pattern’ for some choroideremia patients. Querques et al., (2016) described a similar pattern for geographic atrophy in AMD (Querques *et al*., 2016). These conditions are similarly linked to vast RPE atrophy and visibility of choroid vessels.

## Regional Atrophic Pigment Clumping

In patients with well‐defined regions of atrophy with pigment clumping the pigment seems to form the borders of the atrophy on AO. Figure 1E shows a ring‐shaped atrophy localized superior nasally of the macula. The corresponding OCT scan demonstrates a complete loss of the outer segments. These patterns can increase in their size over time in follow‐ups. The change in Fig. 1E documents a period of 9 months.

To date, limited knowledge is available for examinations with AO retinal cameras in retinal neurodegenerative disease. Other study groups tried to establish patterns of degeneration. (Gale, Feng, Titus, Smith, & Pennesi, 2016). Our findings based on observations of RP patients are of descriptive nature and may represent different stages of photoreceptor degeneration. Further testing and long‐term observations are necessary in a prospective study to confirm our hypotheses and understand the aetiology of these morphological changes.
